# High-performance thermoelectric nanocomposites from nanocrystal building blocks

**DOI:** 10.1038/ncomms10766

**Published:** 2016-03-07

**Authors:** Maria Ibáñez, Zhishan Luo, Aziz Genç, Laura Piveteau, Silvia Ortega, Doris Cadavid, Oleksandr Dobrozhan, Yu Liu, Maarten Nachtegaal, Mona Zebarjadi, Jordi Arbiol, Maksym V. Kovalenko, Andreu Cabot

**Affiliations:** 1 Department of Chemistry and Applied Biosciences, Institute of Inorganic Chemistry, ETH Zürich, Vladimir Prelog Weg 1, CH-8093 Zurich, Switzerland; 2 Laboratory for Thin Films and Photovoltaics, Empa-Swiss Federal Laboratories for Materials Science and Technology, Dübendorf, Überlandstrasse 129, CH-8600 Dübendorf, Switzerland; 3 Advanced Materials Department, Catalonia Energy Research Institute - IREC, Sant Adria de Besos, Jardins de les Dones de Negre n.1, Pl. 2, 08930 Barcelona, Spain; 4 Department of Advanced Electron Nanoscopy, Catalan Institute of Nanoscience and Nanotechnology (ICN2), CSIC and The Barcelona Institute of Science and Technology, Campus UAB, Bellaterra, 08193 Barcelona, Spain; 5 Paul Scherrer Institute, 5232 Villigen PSI, Switzerland; 6 Department of Mechanical and Aerospace Engineering, Rutgers University, 98 Brett Rd, Piscataway, New Jersey 08854-8058, USA; 7 Institució Catalana de Recerca i Estudis Avançats, ICREA, Passeig de Lluís Companys, 23 08010 Barcelona, Spain

## Abstract

The efficient conversion between thermal and electrical energy by means of durable, silent and scalable solid-state thermoelectric devices has been a long standing goal. While nanocrystalline materials have already led to substantially higher thermoelectric efficiencies, further improvements are expected to arise from precise chemical engineering of nanoscale building blocks and interfaces. Here we present a simple and versatile bottom–up strategy based on the assembly of colloidal nanocrystals to produce consolidated yet nanostructured thermoelectric materials. In the case study on the PbS–Ag system, Ag nanodomains not only contribute to block phonon propagation, but also provide electrons to the PbS host semiconductor and reduce the PbS intergrain energy barriers for charge transport. Thus, PbS–Ag nanocomposites exhibit reduced thermal conductivities and higher charge carrier concentrations and mobilities than PbS nanomaterial. Such improvements of the material transport properties provide thermoelectric figures of merit up to 1.7 at 850 K.

## Abstract (web summary)

Nanomaterials provide a route to efficient solid-state conversion between thermal and electrical energy. Here, the authors demonstrate that a combination of metal and semiconductor colloidal nanocrystals can produce thermoelectric nanocomposites with high performance.

## Graphical abstract

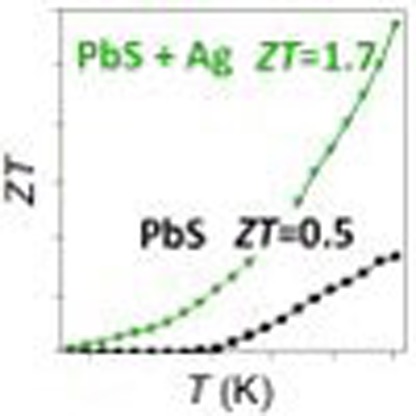

Thermoelectric devices allow direct conversion of heat into electricity and *vice versa*, holding great potential for heat management, precise temperature control and energy harvesting from ubiquitous temperature gradients. The efficiency of thermoelectric devices is primarily governed by three interrelated material parameters: the electrical conductivity, *σ*, the Seebeck coefficient or thermopower, *S*, and the thermal conductivity, *κ*. These parameters are grouped into a dimensionless figure of merit, ZT, defined as ZT=*σS*^2^*Tκ*^−1^ where *T* is the absolute temperature. While there is no known limitation to the maximum thermoelectric energy conversion efficiency other than the Carnot limit, current thermoelectric materials struggle to simultaneously display high σ and *S*, and low *κ*, which prevents their widespread implementation^1^.

Control of the chemical composition and crystallinity of thermoelectric materials at the nanoscale via engineering of multicomponent nanomaterials (nanocomposites) has proven to be effective for the reduction of thermal conductivity by promoting phonon scattering at grain boundaries^2^,^3^,^4^,^5^,^6^,^7^. The remaining major challenge facing the next generation of high-efficiency thermoelectric materials is the enhancement of the thermoelectric power factor (PF=*S*^2^*σ*) while keeping a low thermal conductivity. Strategies to accomplish this goal have focused on increasing the average energy per carrier through energy filtering^8^, carrier localization in narrow bands in quantum confined structures^9^, or the introduction of resonant levels^10^. At the same time, the electrical conductivity must be optimized by properly adjusting the concentration of charge carriers and maximizing their mobility.

These improvements of thermoelectric properties have been mainly demonstrated at the thin-film level, oftentimes using precise but expensive vacuum-based materials growth techniques^11^. However, practical applications of thermoelectric materials demand inexpensive and, for withstanding relatively high temperature gradients, macroscopic devices. In this regard, current approaches to produce bulk nanocomposites, such as ball-milling or the precipitation of secondary phases from metastable solid solutions, lack precision control over the distribution of phases and/or are limited in compositional versatility. Thus, novel cost-effective and general strategies to produce bulk nanocomposites with high accuracy and versatility need to be developed.

Here we demonstrate that a simple route to engineering nanocomposites by the assembly of precisely designed nanocrystal building blocks is able to reach high thermoelectric efficiencies. We propose to blend semiconductor nanocrystals with metallic nanocrystals forming Ohmic contact with the host semiconductor. In such nanocomposites, metallic nanocrystals control charge carrier concentration through charge spill over to the host semiconductor. The goal of this configuration is to reach large charge carrier concentrations without deteriorating the mobility of charge carriers^12^,^13^,^14^,^15^,^16^. In this three-dimensional (3D) modulation doping strategy^17^,^18^,^19^,^20^, composition, size and distribution of semiconductor and metal nanodomains control the nanocomposite transport properties. Consider a slab of a semiconductor sandwiched between two metallic plates. Transfer of charges at the interfaces will cause band bending, extending over charge-screening length. If the width of a semiconductor slab is on the order of this screening length, there will be an overlap of the bended bands and therefore carriers will not be confined to the interfaces, but they will be able to travel through the bulk of a semiconducting region. Similarly, in the 3D case, the size of the semiconducting nanocrystals should be on the order of the screening length, enabling charge transport with minimum scattering. The position of the quasi Fermi level relative to the conduction band of the semiconductor at the metal–semiconductor interface should be adjusted to align the bands and minimize scattering rates. This can be done by selecting metallic nanocrystals of the appropriate material and size and adjusting their volume fraction. The bottom-up approach presented in this work (Fig. 1) offers sufficient materials versatility to harness the key benefits of such 3D modulation doping by selecting materials with appropriate Fermi levels and allowing a facile control of nanocrystals size and volume fraction. For this study, PbS was selected as inexpensive host semiconductor that comprises Earth-abundant elements (that is, contrary to tellurides), and holds great potential for reaching high thermoelectric efficiencies; with reported ZT values of up to 1.3 at 923 K in PbS–CdS^21^. Silver is chosen as a nanoscopic metallic dopant owing to its low work function (4.26–4.9 eV) (refs 22, 23, 24) needed for efficient injection of electrons into the PbS conduction band. The PbS–Ag nanocomposites derived from colloidal PbS and Ag nanocrystals exhibit high electrical conductivities due to (i) injection of electrons from Ag nanoinclusions to the host PbS and (ii) improved charge carrier mobility. The simultaneous combination of high electrical conductivity, relatively large Seebeck coefficients, and reduced thermal conductivities provides thermoelectric figures of merit up to 1.7 at 850 K.

## Results

### PbS–Ag nanocomposites

**Figure 2:**
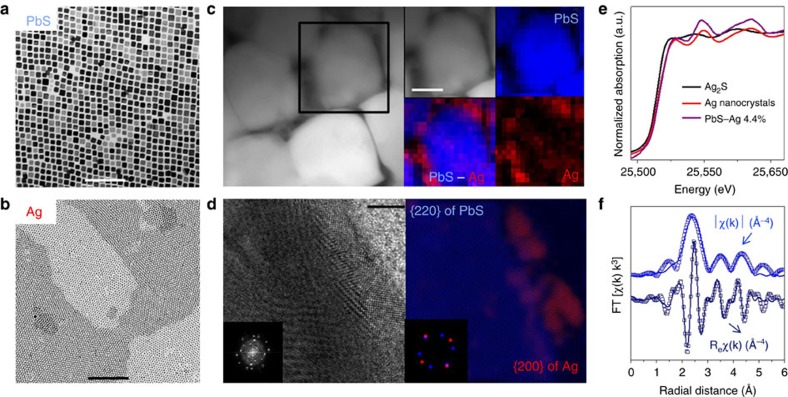
Structural and compositional characterization of initial NCs and resulting PbS–Ag 4.4 mol% nanocomposite. TEM micrographs of (**a**) PbS and (**b**) Ag NCs; (**c**) HAADF-STEM micrograph and elemental EDX maps; and (**d**) HRTEM micrograph of the PbS–Ag interface with the corresponding power spectrum (inset) and filtered colourful composite image for the {220} family of planes of PbS (blue) and {200} family of planes of Ag (red). In the filtered images, PbS and Ag are visualized along their [111] and [001] zone axes, respectively (Supplementary Note 1); (**e**) Ag K-edge XANES spectra of Ag_2_S reference (black), Ag NCs (red) and PbS–Ag 4.4 mol% nanocomposite (purple); (**f**) Fourier transform magnitude, |χ_(k)_|, and real part, _Re_χ_(k)_, of the Pb L_3_-edge EXAFS spectrum, the experimental data are given by the dotted line, the best fit by the solid line. Scale bars, 100 nm (**a**), 100 nm (**b**), 50 nm (**c**), 5 nm (**d**).

PbS–Ag nanocomposites were produced by combining cubic PbS nanocrystals (*ca.* 11 nm, Fig. 2a) and spherical Ag nanocrystals (*ca.* 3 nm Fig. 2b), followed by the evaporation of a solvent. Thereby obtained powdered nanocrystal blend was annealed to remove residual organic compounds and then hot-pressed into pellets. This simple procedure yielded nanocomposites with a highly homogeneous distribution of metallic Ag nanodomains at the interfaces of PbS grains, as evidenced by high-resolution transmission electron microscopy (HRTEM), high-angle annular dark field scanning transmission electron microscopy and energy-dispersive X-ray spectroscopy (Fig. 2c,d). Further atomistic insights into bonding and chemical identities of constituents were obtained by X-ray absorption spectroscopy (XAS) at the Ag K edge (25515, eV) and Pb L_3_ edge (13035, eV), respectively. Linear combination fitting of the X-ray absorption near edge structure (XANES) around the Ag absorption K edge, using references of Ag_2_S and Ag metal, confirmed that at least 97% of Ag retained its metallic state (Fig. 2e, Supplementary Fig. 1 and Table 1). Only a small percentage of Ag may had diffused as Ag^+^ within the PbS matrix. In addition, fitting of the Pb L_3_ edge extended X-ray absorption fine structure spectra indicated interatomic distances and coordination numbers characteristics of PbS^25^ (Fig. 2f). Metallic lead, lead oxide or lead sulfate species did not noticeably contribute to the spectra, suggesting that the presence of these phases can be disregarded (Supplementary Table 2).

### Thermoelectric properties

**Figure 3:**
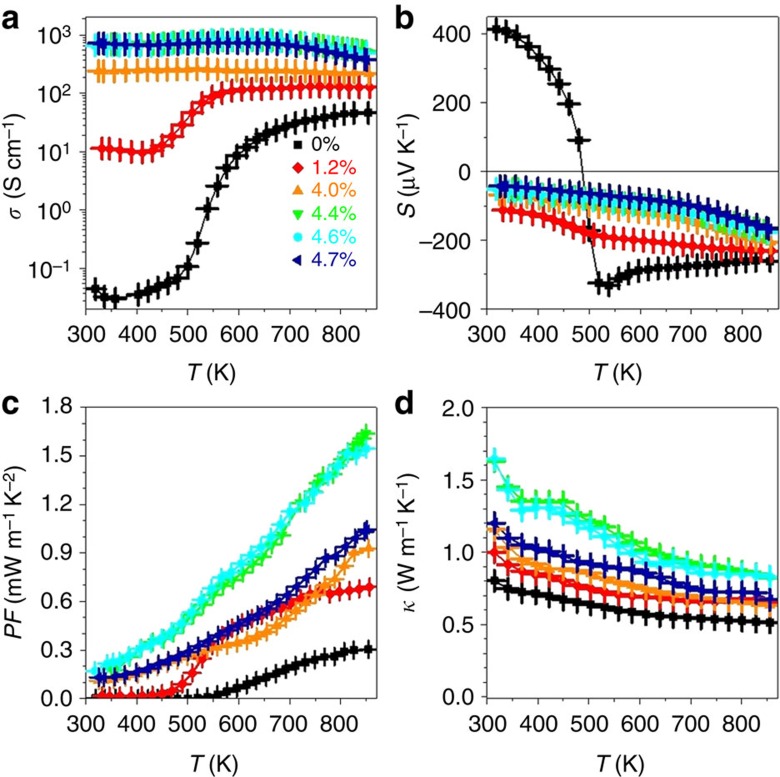
Thermoelectric characterization of PbS–Ag nanocomposites. Temperature dependence of the (**a**) electrical conductivity, σ; (**b**) Seebeck coefficient, *S*; (**c**) thermal conductivity, κ; and (**d**) power factor, PF. Error bars were estimated from the repeatability of the experimental result; 3–5 measurements were carried out for each material.

To determine the effect of the Ag content on thermoelectric performance, a series of nanocomposites with Ag concentrations up to 5 mol% were prepared and analysed. Ag-free PbS nanomaterials exhibited relatively low electrical conductivities, which greatly increased from 0.07 S cm^−1^ at room temperature (RT) up to 46 S cm^−1^ at 850 K due to band to band charge carrier thermal excitation (Fig. 3a). This increase was accompanied by a sign inversion of the Seebeck coefficient from positive to negative at around 470 K (Fig. 3b). On the contrary, PbS–Ag nanocomposites possess significantly higher, Ag concentration-dependent, electrical conductivities. At RT, electrical conductivities of up to 660 S cm^−1^ were measured for Ag concentrations above 4 mol% (Fig. 3a). Furthermore, over the whole studied temperature range of 300–850 K, PbS–Ag nanocomposites exhibit negative Seebeck coefficients. Unlike to electrical conductivity, Seebeck coefficient decreases with increasing Ag content. Hence an optimal concentration of *ca.* 4.4–4.6 mol% Ag nanocrystals was established for maximizing the PF (Fig. 3c). Overall, around 20 PbS–Ag pellets were produced, all showing PFs above 1 mW m^−1^ K^−2^ at 850 K, with a champion value of 1.68 mW m^−1^ K^−2^ at 850 K for a PbS–Ag 4.4 mol%. This is a sixfold increase over identically prepared Ag-free samples, and a significant improvement by 23–47% over previously reported PbS-based nanocomposites^21^,^26^.

The thermal conductivities of the PbS–Ag nanocomposites monotonically increased with the Ag content (Fig. 3d) due to the increase of the electronic contribution (Supplementary Fig. 2 and Methods), yet not exceeding in the high-temperature range the values reported for PbS-based nanocomposites produced by co-precipitation of secondary phases^21^,^26^,^27^. Thus, beyond injecting charge carriers and facilitating charge transport between PbS nanocrystals, Ag nanodomains also assisted in blocking phonon propagation. The relatively low thermal conductivities may also in part result from phonon scattering at nanodomains within the large PbS grains as observed by extensive HRTEM analysis (Supplementary Figs 3 and 4 and Note 2).

**Figure 4:**
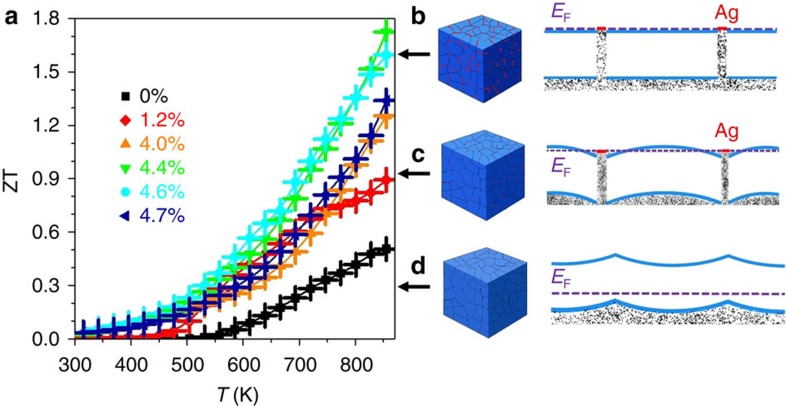
Figure of merit and schematic representation of the electron energy band alignment. (**a**) Figure of merit, ZT, of PbS and PbS–Ag pellets; (**b**) band alignment in PbS–Ag nanocomposite with a larger amount of Ag and flat bands across the whole PbS domains; (**c**) band aligment in PbS–Ag nanocomposite with a low Ag volume fraction, showing electron energy wells in between PbS domains; (**d**) band alignment in bare PbS with an upward band-bending at the PbS intergrains. Error bars were estimated from the repeatability of the experimental result; 3–5 measurements were carried out for each material.

Overall, the outstanding electrical properties along with low thermal conductivities of PbS–Ag samples resulted in thermoelectric figures of merit of up to ZT=1.7 at 850 K (Fig. 4a). This value corresponds to a 30% increase over the highest figure of merit obtained for PbS to date (ZT=1.3, Pb_0.975_Na_0.025_S+3% CdS)^21^ and is comparable to the highest thermoelectric figures of merit reported for other lead chalcogenide materials (Supplementary Table 3)^3^,^21^,^26^,^27^,^28^,^29^,^30^.

## Discussion

**Figure 5:**
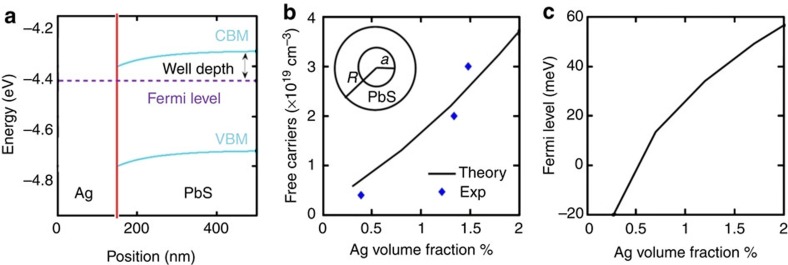
Theoretical calculations. (**a**) Band alignment at the Ag-PbS interface considering bulk Ag and bulk PbS at 300 K. The Fermi level is pinned at −4.4 eV (silver work function). Electrons in PbS experience a well, as marked, when going to the silver side. (**b**) Carrier concentration versus silver volume fraction. Experimentally measured Hall data are shown by blue dots. Free carrier concentration, calculated using Anderson model and Poisson solver for the geometry shown in the inset of the figure, is shown by solid line. The inner sphere represents a silver NC (*a*=1.5 nm) and the outer sphere represent PbS host matrix with radius *R* (silver volume fraction=(*a*/*R*)^3^. (**c**) Fermi level plotted with respect to the conduction band minimum of PbS (far away from the interface). When negative, this value corresponds to the well depth experienced by electrons when moving between PbS grains.

The high PFs displayed by PbS–Ag nanocomposites are at the origin of their outstanding thermoelectric figures of merit. Such high PFs could not be explained by a simple weighed sum of the properties of two randomly distributed compounds (Supplementary Table 4 and Supplementary Discussion). Neither can we explain the high electrical conductivity by percolation transport through Ag domains, as much lower Seebeck coefficients would be expected for a metallic conductor. It must be also pointed out that doping of PbS with Ag^+^ ions cannot explain these transport properties either, since previous studies have demonstrated Ag^+^ to be a p-type dopant for PbS^31^,^32^,^33^,^34^. Simultaneous combination of high electrical conductivity and relatively large Seebeck coefficients can be explained by an efficient injection of electrons from the metal to the conduction band of the semiconductor (Fig. 4b–d). In this regard, RT Hall charge carrier concentration measurements evidenced an increase in concentration of majority charge carriers from *p*=1 × 10^16^ in the bare PbS nanomaterial to *n*=3 × 10^19^ in PbS–Ag 4.4 mol% samples (Fig. 5 and Supplementary Table 5). This is consistent with the initial Ag Fermi level above that of the intrinsic PbS. What is surprising is that the obtained charge carrier mobilities also increased with the Ag introduction, from 20 cm^2^ V^−1^ s^−1^ for bare PbS to 90 cm^2^ V^−1^ s^−1^ for PbS–Ag 4.4 mol% (Supplementary Table 5). In a simple modulation doping scenario, one would expect the injection of charge carriers from Ag nanodomains into PbS to have little negative impact on the charge carrier mobility. However, in PbS–Ag nanocomposites, the actual effect of Ag was found to facilitate charge transport through the material. We attribute this to a reduction of the energy barriers between PbS crystal domains (Fig. 4b–d). To determine the band alignment, we used the Anderson model to align the vacuum levels of Ag and PbS and then solve the Poisson equation self consistently assuming a parabolic two-band model for PbS (Fig. 5a). We used the Ag work function, which depends on its crystallographic surface and domain size, as a fitting parameter to fit the experimentally measured Hall data (Fig. 5b). The obtained fitted parameter was 4.4 eV, which is in the correct range (4.26–4.9 eV) (refs 22, 23).

At a bulk metal–semiconductor junction, the Fermi level is pinned by the metallic layer due to the large carrier density in the metallic layer (Fig. 5a). However, for small nanocrystals, the number of electrons is limited and at low metal concentrations it may be not enough to completely pin the Fermi level. To simulate charge transfer from Ag nanocrystals to PbS, we assumed Ag nanocrystals were spheres of radius 1.5 nm (to replicate the 3-nm size of Ag nanodomains observed) embedded within another sphere made out of PbS. The radius of PbS sphere was determined by the volume fraction of each sample (Fig. 5b, inset). Figure 5c shows the Fermi level with respect to the bottom of the conduction band. This is an indicator for the effective well depth for the electrons as marked in Fig. 5a. As can be seen in Fig. 5c, when the Ag fraction increases, the Fermi level also increases, lowering the effective well depth in between PbS grains, which reaches zero at around a 0.5 % Ag volume fraction, that is, 1.5 mol%. At this point and beyond, there is no effective well in the path of the electrons (Fig. 4b). Therefore, as the Ag fraction increases, the electron-nanocrystal scattering decreases, which is consistent with the observed enhancement in electron mobility.

**Figure 6:**
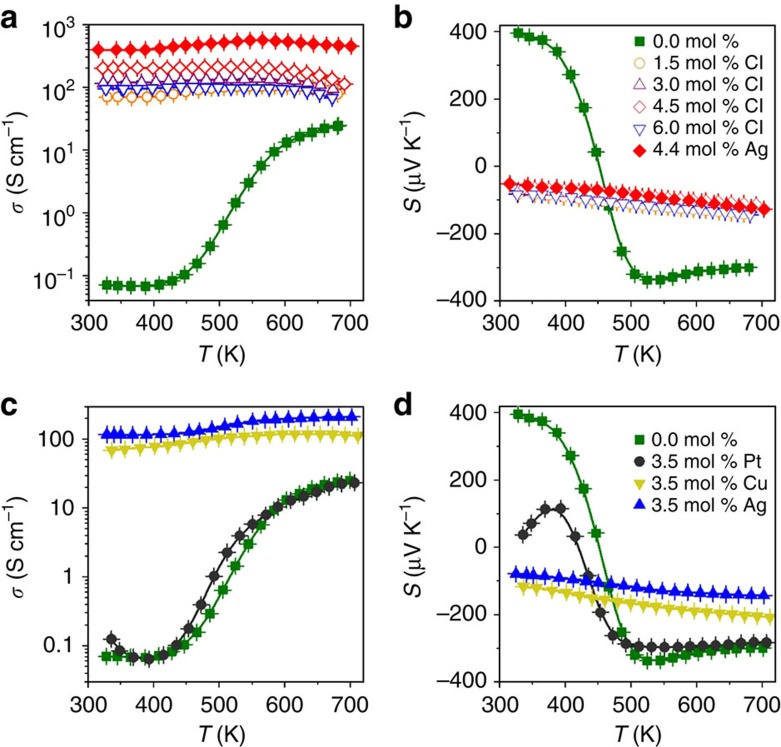
Electrical properties of PbS nanomaterials with different doping strategies. (**a**) σ and (**b**) *S* of *x* mol% PbS (*x*=1.5, 3.0, 4.5 and 6.0) doped with Cl (open symbols) compared with pure PbS (0.0 mol %) and PbS–Ag (4.4 mol%). (**c**) σ and (**d**) *S* of PbS (0.0 mol%) and PbS-X (3.5 mol%) with X=Pt, Cu and Ag.

Note that the high PFs found in PbS–Ag nanocomposites cannot be obtained by a conventional doping strategy, where the introduction of ionic impurities would lead to increased scattering of charge carriers. This is shown in Fig. 6a,b, where the thermoelectric properties of PbS nanomaterials with different chlorine concentrations are presented. By introducing Cl^−^ ions, a common dopant used in n-type PbS, the electron density can be increased, which translates into an enhancement of the electrical conductivity^35^. However, the highest electrical conductivities reached by halide doping remained below the values obtained for PbS–Ag nanocomposites. Thus, despite that PbS:Cl (4.5 mol%) nanomaterials had charge carrier concentrations in the same order of magnitude and similar Seebeck coefficients as the PbS–Ag (4.4 mol%) nanocomposites, the later showed significantly higher electrical conductivities and, therefore, higher PFs.

Besides PbS–Ag nanocomposites, a variety of other compositions were accomplished from colloidal nanocrystal building blocks, often showing similar synergistic effects on charge transport. For instance, we have carefully examined composites of PbS with nanoscopic Cu (Supplementary Fig. 5) and Pt (Supplementary Fig. 6). Like Ag, Cu has a low work function and hence is able to inject charges into the conduction band of PbS. On the contrary, Pt has a much higher work function and does not exhibit efficient charge transfer (Fig. 6c,d).

Colloidal nanocrystals can be prepared with unmatched control over size, composition, shape, crystal phase and surface chemistry and benefit from facile handling and mixing in stable dispersions^36^. The current availability of a rich palette of such building blocks lends the opportunity to create a plethora of different nanocomposites by simply blending nanocrystals of various materials in the appropriate proportions and, afterward, consolidating them into arbitrarily shaped composites. Thus the facile bottom–up approach used here allows engineering a nearly endless variety of nanocomposites, which will allow a high-throughput screening of materials in the effort to maximize the thermoelectric energy conversion efficiency of durable, silent and scalable thermoelectric devices.

## Methods

### Nanocomposite preparation

*Chemicals*. Lead(II) oxide (PbO, 99.9%), copper(I) acetate (CuOAc, 97%), silver nitrate (AgNO_3_, ≥99.8%), iron(III) nitrate nonahydrate (Fe(NO_3_)_3_·9H_2_O, 99.99%), platinum acetylacetonate (Pt(acac)_2_, 97%), manganese(0) carbonyl (Mn_2_(CO)_10_, 98%), elemental sulfur (99.998%), oleic acid (OA, tech. 90%), 1-octadecene (ODE, 90%), oleylamine (OLA, tech. 70%) and benzyl ether (BE, 98%) were purchased from Aldrich. Tri-n-octylamine (TOA, 97%) was purchased from Across. Tetradecylphosphonic acid (TDPA, 97%) was purchased from PlasmaChem. Hexane, toluene, ethanol, anhydrous chloroform and anhydrous methanol were obtained from various sources. All chemicals, except OLA, were used as received without further purification. OLA was distilled to remove impurities.

*Synthesis of nanocrystals*. All syntheses were carried out using standard air-free techniques; a vacuum/dry argon Schlenk line was used for synthesis and an argon-filled glove box for storing and handling air and moisture-sensitive chemicals.

PbS nanocrystals with a mean edge size of 11 nm were prepared similarly to previously reported procedures^35^. In a typical synthesis, PbO (4.46 g, 20 mmol) and OA (50 ml, 0.158 mol) were mixed with 100 ml of ODE. This mixture was degassed at RT and 100 °C for 0.5 h each to form the lead oleate complex. Then the solution was flushed with Ar, and the temperature was raised to 210 °C. At this temperature, a sulfur precursor, prepared by dissolving elemental sulfur (0.64 g, 20 mmol) in distilled OLA (20 ml, 0.061 mol), was rapidly injected. The reaction mixture was maintained between 195 °C and 210 °C for 5 min and then quickly cooled down to RT using a water bath. Ag nanocrystals with an average diameter of 2–3 nm were produced using a modified approach of that reported by Wang *et al.*^37^ In a typical reaction, AgNO_3_ (0.17 g, 1 mmol), Fe(NO_3_)_3_·9H_2_O (0.04 g, 0.01 mmol), OA (10 ml, 0.0316, mol) and OLA (10 ml, 0.0305, mol) were mixed and placed under Ar at RT for 30 min. Afterwards the reaction mixture was heated to 120 °C at the rate of 5 °C min^−1^ and kept at this temperature for an additional 60 min. Cu nanocrystals with an average diameter of 5–6 nm were prepared following the approach developed by Yang *et al.*^38^ In a typical synthesis, TOA (50 ml, 0.114 mol) was heated in a 100 ml three-neck flask to 130 °C for 30 min under Ar atmosphere. After cooling to RT, CuOAc (613 mg, 5 mmol) and TDPA (696 mg, 2.5 mmol) were added to the flask. The mixture was heated to 180 °C and maintained at this temperature for 30 min. Then, the reaction temperature was further increased to 270 °C and held for another 30 min. Cu nanocrystals are highly air sensitive and easily oxidized. To avoid any possible oxidation, the nanocrystals were purified in an Ar filled glove box. Pt nanocrystals with an average diameter of 6 nm were prepared using the method developed by Murray *et al.*^39^ In a typical synthesis, Pt(acac)_2_ (80 mg, 0.20 mmol) was dissolved in BE (10 ml, 52.6 mmol), OLA (7.36 ml, 22.37 mmol) and OA (1.25 ml, 3.94 mmol) under Ar atmosphere for 30 min at 60 °C. The precursor mixture was heated to 160 °C and a solution of 80 mg Mn_2_(CO)_10_ in 1 ml of chloroform was rapidly injected. Afterwards, the temperature was heated to 200 °C and held for an additional 30 min at this reaction temperature. Finally, the crude solution was cooled to RT.

*Blending of nanocrystals*. In this work we prepared PbS-metal semiconductor nanocomposites with different metal concentrations. The blending of nanocrystals was performed by wetting 1 g of dried PbS nanocrystals (a powder) with different amounts of a 0.093 M solution of metallic nanocrystals in anhydrous chloroform. Subsequently, the solvent was allowed to evaporate under Ar atmosphere. The concentration of metallic nanocrystals in chloroform was initially estimated by mass (considering a 30% of organic ligand) and later verified by inductively coupled plasma (ICP) spectroscopy. The values were found to differ slightly between the estimation from the weight and from ICP. The final quantities reported in this work correspond to the values obtained from ICP measurements.

*Pellet fabrication*. Before powder consolidation in a hot press, the nanocrystal powders were treated thermally to decompose the remaining organic ligands present at the nanocrystal surface. All nanocrystal powders were heated to 450 °C at 10 °C min^−1^ and held at this temperature for 1 h under an Ar flow. After cooling to RT, the nanocrystal powders were pressed using a custom-made hot press. In this system, heat is provided by an induction coil operated in the RF range and applied directly to a graphite die acting as a susceptor. Before hot pressing, coarse powders were ground into fine powder using a mortar inside the glove box and then loaded into a 10-mm diameter graphite die lined with 0.13-mm thick graphite paper. The filled die was placed in the hot press system. The densification profile applied an axial pressure of 45 MPa before heating the die to between 420 and 440 °C. The temperature was held between 420 and 440 °C for 4 min. The pressure was then removed and the die cooled to RT. The resulting pellets were >85% dense compared with theoretical maximum into air-stable monoliths measuring ∼1-mm thick by 10 mm in diameter. The density of the pressed pellets was measured by the Archimedes method.

### Structural characterization

The size and shape of the initial nanocrystals were examined by TEM using a ZEISS LIBRA 120 instrument, operating at 120 kV. Structural and compositional characterizations of the nanocomposites were examined after thermoelectrical characterization. TEM Samples were mechanically thinned to 20–30 μm and further thinned to electron transparency by Ar^+^ polishing using a Gatan Precision Ion Polishing System. HRTEM and scanning transmission electron microscopy studies were conducted by using a FEI Tecnai F20 field emission gun microscope operated at 200 kV with a point-to-point resolution of 0.19 nm, which is equipped high-angle annular dark field and energy-dispersive X-ray spectroscopy detectors. ICP atomic emission spectrometry was used for elemental analysis of the nanocomposites, especially to determine the ratio between Pb and Ag, Pt or Cu. ICP atomic emission spectrometry measurements were carried out using Perkin–Elmer Optima instrument, model 3200RL, under standard operating conditions. Samples were prepared by microwave-assisted digestion of the dried materials in a mixture of HNO_3_ and H_2_O_2_ in a closed container. X-ray powder diffraction analyses were collected directly on the as-synthesized nanocrystals and final pellets using a Bruker AXS D8 Advance X-ray diffractometer with Ni-filtered (2 μm thickness) Cu K_α_ radiation (*λ*=1.5406 Å) operating at 40 kV and 40 mA (Supplementary Fig. 7). A LynxEye linear position-sensitive detector was used in reflection geometry. XAS measurements were carried out at the X10DA (SuperXAS) beamline at the Swiss Light Source, Villigen, Switzerland, which operated with a ring current of ∼400 mA in top-up mode. The polychromatic radiation from the superbend magnet, with a magnetic field of 2.9 T and critical energy of 11.9 keV, was monochromatized using a channel cut Si(311) crystal monochromator. Spectra were collected on pressed pellets optimized to 1 absorption length at the Ag K edge (25515, eV) and Pb L_3_ edge (13035, eV) in transmission mode. XAS data were treated with the Demeter software suite^40^. For all samples, three spectra were acquired and merged. These averaged XAS data were background-subtracted and normalized. Linear combination fitting of the Ag K edge X-ray absorption near edge structure spectra was performed over the energy range from 25,498 to 25,598 eV. The goal was to determine composition of Ag-species present in the PbS–Ag sample. For this, reference spectra of metallic Ag NPs and Ag_2_S were combined linearly. Fourier transformation of the Pb L_3_ edge extended X-ray absorption fine structure spectra was performed over the k range of 3−9 Å^−1^ yielding a pseudo radial structure function. The R-range from 1 to4.8 Å was fitted using theoretical single scattering paths of bulk PbS (S6)^25^ based on crystallographic data.

### Thermoelectric characterization

*Electric properties*. The pressed samples were polished, maintaining the disk-shape morphology. Final pellets had a 10-mm diameter and were ∼1 mm thick. The Seebeck coefficient was measured using a static DC method. Electrical resistivity data were obtained by a standard four-probe method. Both the Seebeck coefficient and the electrical resistivity were simultaneously measured with accuracies better than 1% in a LSR-3 LINSEIS system from RT to 850 K, under helium atmosphere. Samples were held between two alumel electrodes and two probe thermocouples with spring-loaded pressure contacts. A resistive heater on the lower electrode created temperature differentials in the sample to determine the Seebeck coefficient. Samples measured up to 850 K were spray coated with boron nitride to minimize out-degassing except where needed for electrical contact with the thermocouples, heater and voltage probes. In addition, before high-temperature measurements, samples were heated within the LINSEIS system in a He atmosphere up to 850 K at 3 K min^−1^ and hold at this temperature for 10 min with the boron nitride coating. Such preliminary treatment warrants sample stability for all the cycles tested (Supplementary Figs 8 and 9 and Discussion). Carrier concentration and mobility were estimated from Hall Effect measurements, which were performed at RT using an Ecopia HMS-3000 set-up with golden spring-loaded contacts positioned at the edges of plates in the Van der Pauw configuration.

*Thermal properties*. An XFA 600 Xenon Flash Apparatus was used to determine the thermal diffusivities of all samples with an accuracy of *ca.* 6%. Total thermal conductivity (κ) was calculated using the relation κ=*DC*_p_*ρ*, where *D* is the thermal diffusivity, *C*_p_ is the heat capacity and *ρ* is the mass density of the pellet. The *ρ* values were calculated using the Archimedes method. The specific heat (C_p_) of the samples was measured using a Differential Scanning Calorimeter DSC 204 F1 Phoenix from NETZSCH (Supplementary Fig. 10). The electronic contribution of the thermal conductivity was calculated using the Wiedemann–Franz law (Supplementary Fig. 11 and Methods).

## Additional information

**How to cite this article**: Ibáñez, M. *et al.* High-Performance Thermoelectric Nanocomposites from Nanocrystal Building Blocks. *Nat. Commun.* 7:10766 doi: 10.1038/ncomms10766 (2016).

## Supplementary Material

Supplementary Information Supplementary Figures 1-11. Supplementary Tables 1-5, Supplementary Notes 1-2, Supplementary Discussion, Supplementary Methods and Supplementary References.

## Figures and Tables

**Figure 1:**
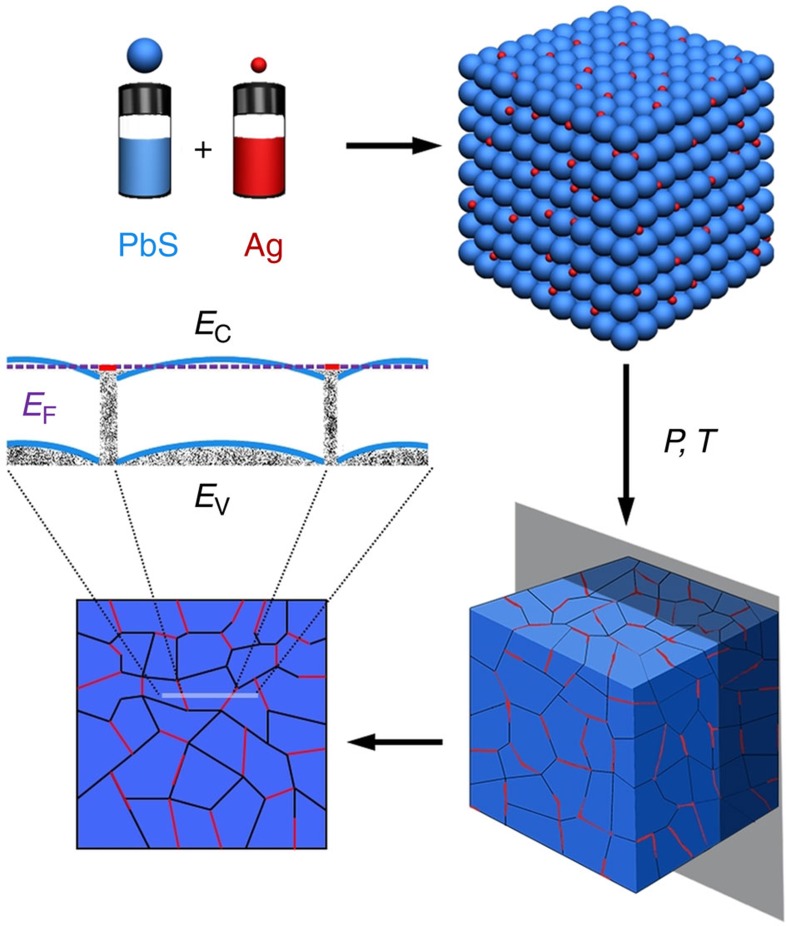
Bottom-up Design. Bottom-up assembly process to produce PbS–Ag TE nanocomposites from the assembly of PbS (blue) and Ag (red) NCs, and the corresponding band alignment of the resulting nanocomposite.
