# Supplementary material for: High-performance thermoelectric nanocomposites from nanocrystal building blocks
Source: Nat Commun. 2016 Mar 7;7:10766. doi: 10.1038/ncomms10766 (PMC4786643; doi:10.1038/ncomms10766)
Supplement: Supplementary Information — Supplementary Figures 1-11. Supplementary Tables 1-5, Supplementary Notes 1-2, Supplementary Discussion, Supplementary Methods and Supplementary References. [file ncomms10766-s1.pdf]

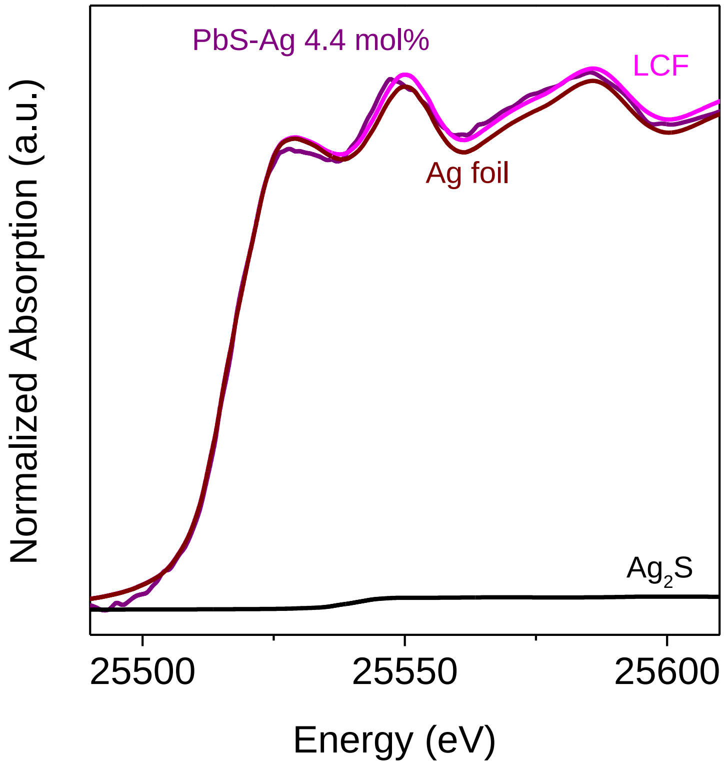

Supplementary Figure 1. Linear combination fit of 4.4% Ag-PbS with the weighted components and the residual error.

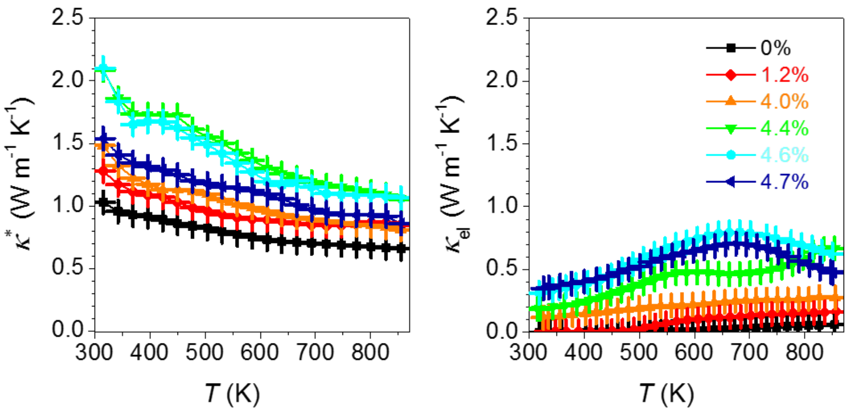

Supplementary Figure 2. Temperature dependence of the porosity corrected thermal conductivity (κ^*) and the electronic contribution (κ_e) for x mol% PbS-Ag with x=0 (black squares), 1.2 (red diamonds), 4.0 (orange triangles up), 4.4 (green triangles down), 4.6 (cyan cercles) and 4.7 (blue triangles left) up to 850 K.

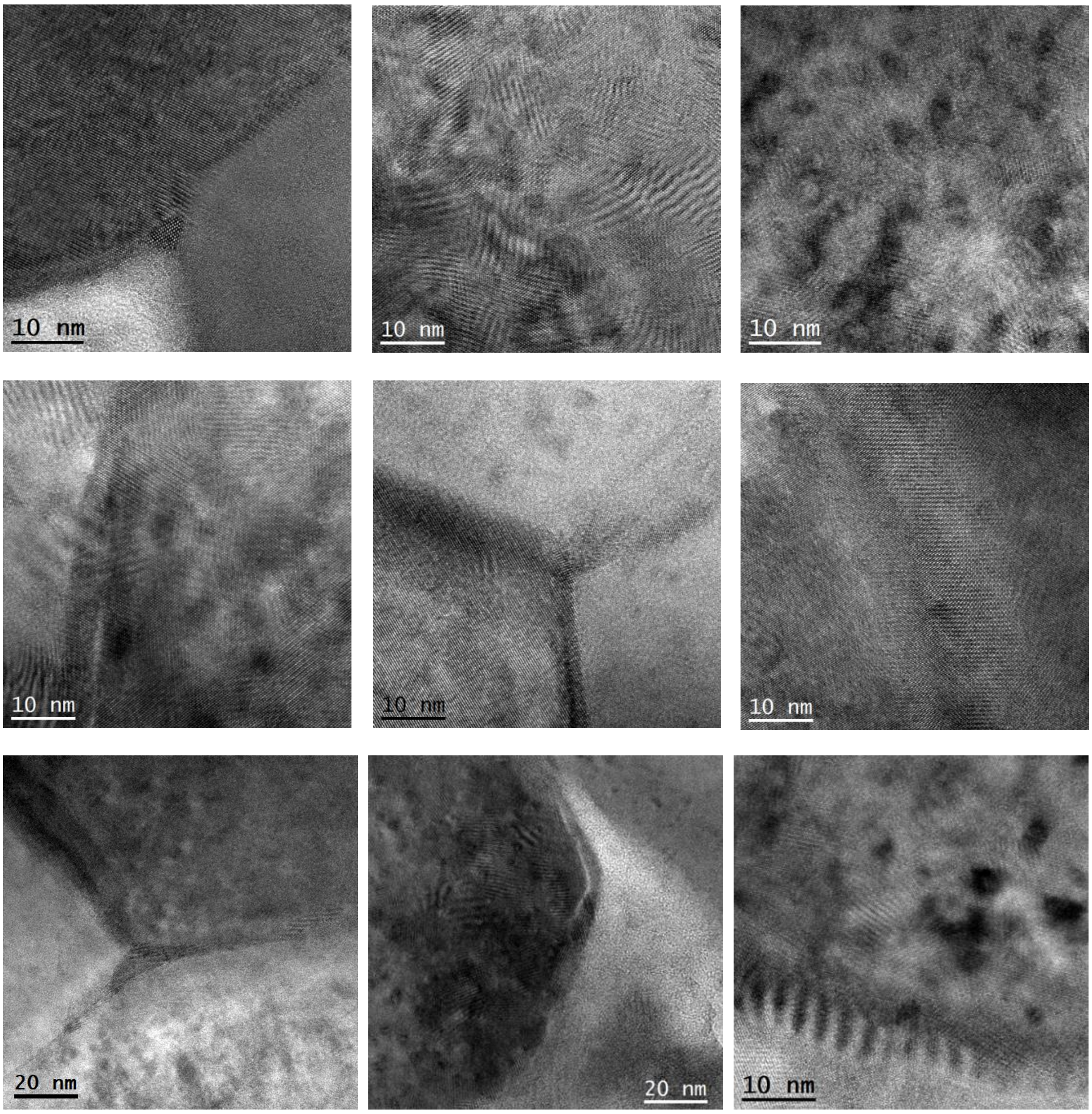

Supplementary Figure 3. Selection of HRTEM images showing regions with different crystallographic phases along the grain boundaries and inclusions inside the grains.

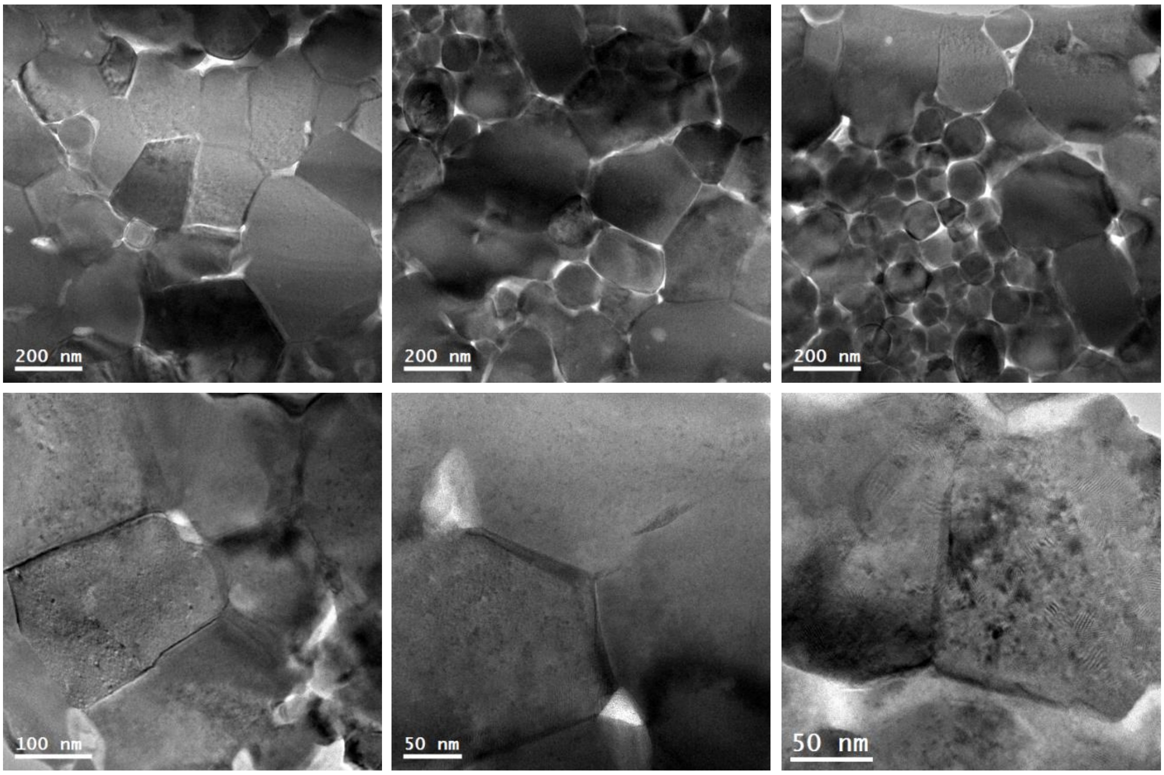

Supplementary Figure 4. Selection of TEM images showing the microstructure of the PbS bulk sample in different directions.

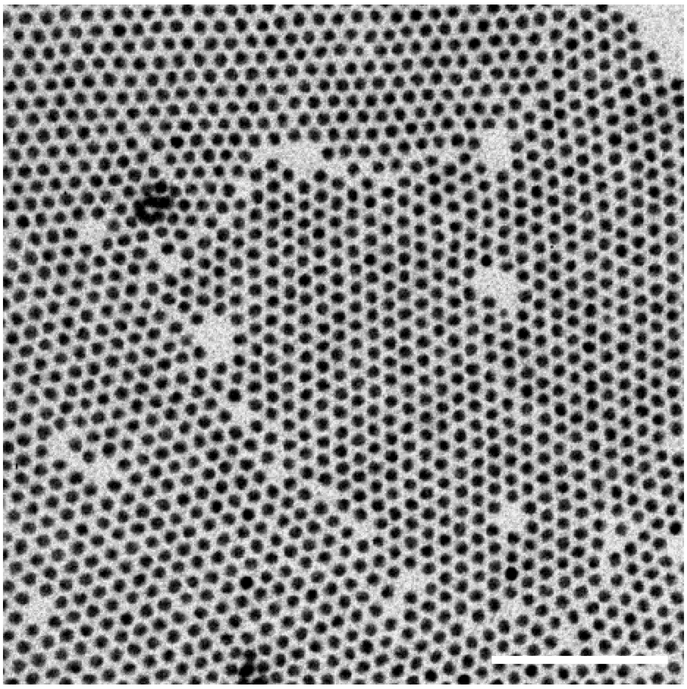

Supplementary Figure 5. TEM image of Cu nanocrystals. Scale bar is 100 nm.

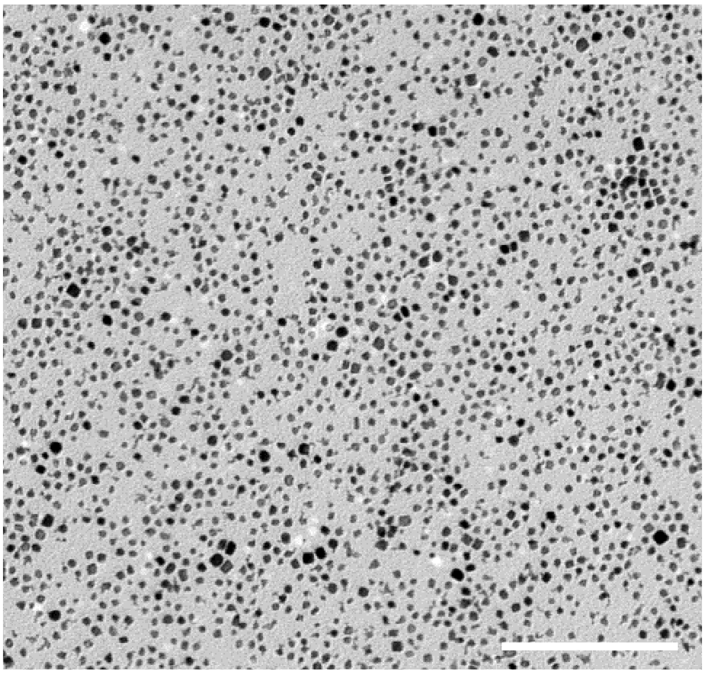

Supplementary Figure 6. TEM image of Pt nanocrystals. Scale bar is 100 nm.

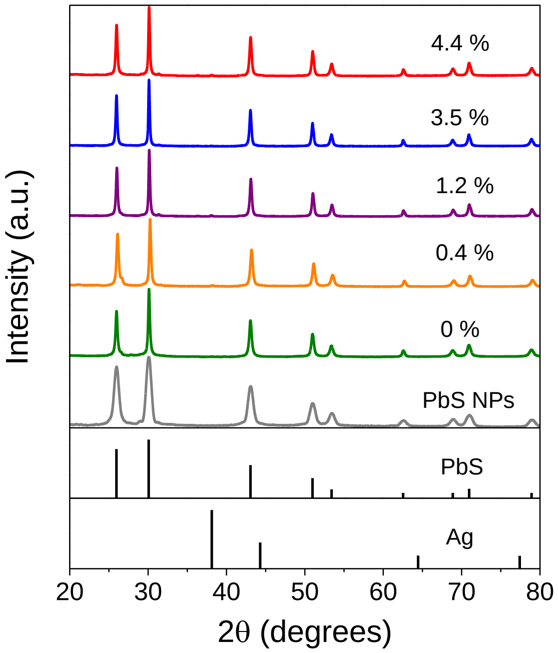

Supplementary Figure 7. XRD patterns of PbS NCs (grey) and x mol% PbS-Ag nanocomposites with x=0 (green), 0.4 (orange), 1.2 (purple), 3.5 (blue), 4.4 (red). The reference patterns for PbS (galena) and Ag are shown as black lines.

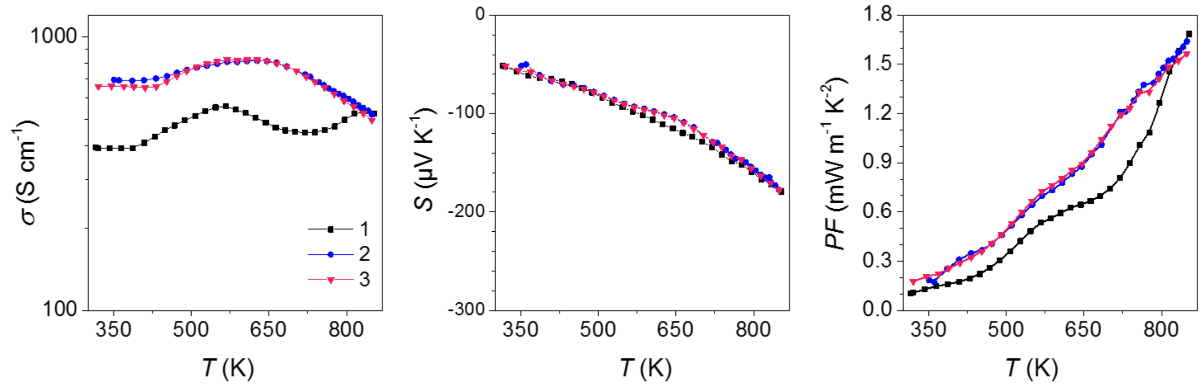

Supplementary Figure 8. Temperature dependence of the electrical conductivity, Seebeck coefficient and power factor of a 4.4 mol% PbS-Ag nanocomposite measured 3 consecutive times during heating up to 850 K. No pre-stabilization treatment was carried out before the first measurement.

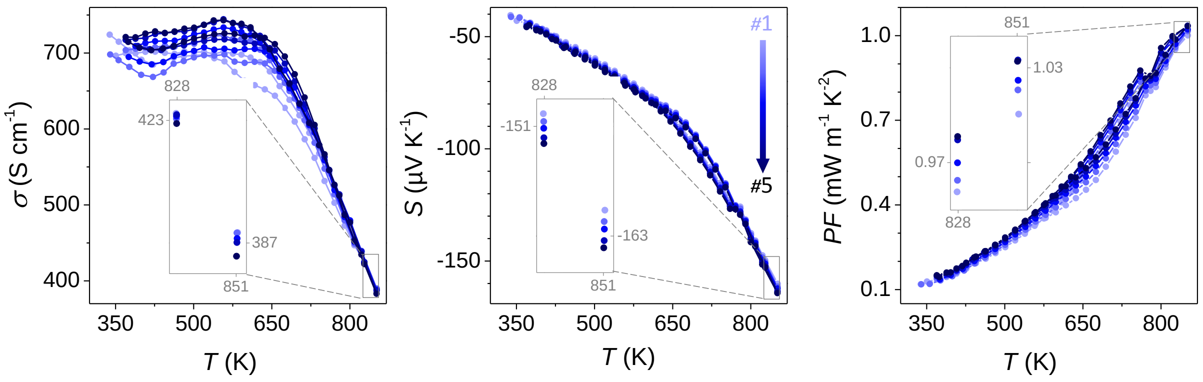

Supplementary Figure 9. Temperature dependence of the electrical conductivity, Seebeck coefficient and power factor of a 4.7 mol% PbS-Ag nanocomposite measured for 5 consecutive temperature cycles up to 850 K.

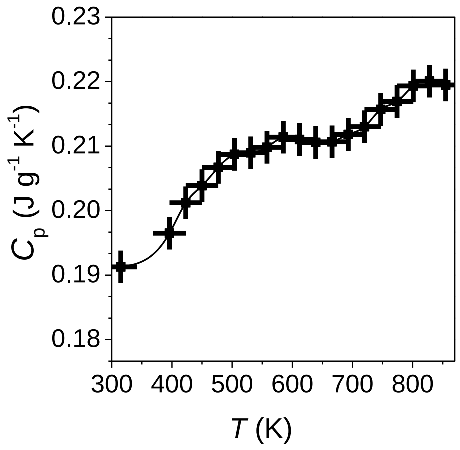

Supplementary Figure 10. C_p of the PbS nanomaterial measured using DSC

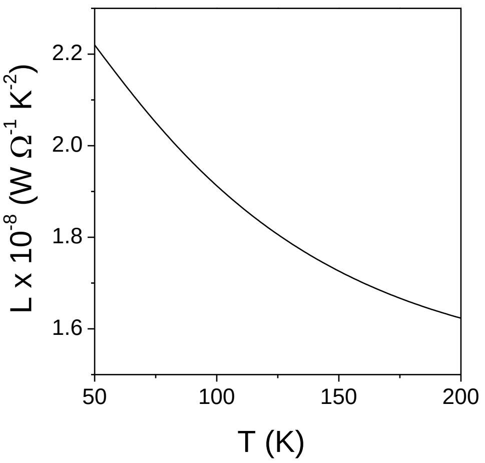

Supplementary Figure 11. The Lorentz number estimation based on the Seebeck coefficient measurements and assuming scattering from acoustic phonons, following Fermi-Dirac statistics.

Supplementary Table 1. Linear combination fit results for 4.4% Ag-PbS. Summary of the results of the linear combination fit for 4.4 mol% Ag-PbS nanocomposite. The remaining deviation can be explained by the oxidation of silver during sample preparation in air for the XAS measurements. R-factor: 0.0018; Reduced chi-square: 0.00025.

**Reference spectrum**	**Weight**	**E_0 shift (eV)**
Ag NPs	0.100	0.772
Ag_2S	0.027	25.068

Supplementary Table 2: Best fit parameters for 4.4% Ag-PbS. CNs were set to the bulk values when performing the fit. The first shell consisting of S atoms was fitted first using a range from 1 to 3.1 Å assuming the coordination number (*CN*) to be the same as in the bulk [24]. The fitted parameters for the first shell (amplitude reduction factor, S_0^2, energy shift, ΔE_0, bond distance difference, ΔR, and Debye-Waller factor, σ^2) were maintained constant when fitting the second shell while extending the range to 4.8 Å. The *CN* of the second shell was set to 12 and ΔE_0 was assumed to be the same for both shells. Reduced chi-square: 476; R-factor: 0.0214 (The reduced chi-square and the *R* factor are defined according to the FEFFIT conventions (ref: http://cars9.uchicago.edu/ifeffit/Documentation).

**Scattering Path**	**CN**	**$S0^2$**	**ΔE_0 [eV]**	**R [Å]**	**σ^2 [$Å^2$]**
Pb-S	6.0	0.63 ± 0.09	2.8 ± 1.1	2.95± 0.02	0.021 ±0.003
Pb-Pb	12.0	0.62 ± 0.12	2.8 ± 1.1	4.20 ±0.01	0.020 ± 0.004

Supplementary Table 3. Table with the thermoelectric properties and corresponding figures of merit of state of the art lead chalcogenides.

**System**	σ **(S cm^{-1})**	***S*** **(μV K^{-1})**	***PF*** **(μW cm^{-1} K^{-2})**	κ **(W m^{-1} K^{-1})**	ZT_{max}	T_{ZTmax} **(K)**	**Ref.**
$Pb_{0.975}Na_{0.025}S$ + 3.0% CdS	142	280	11.1	0.79	1.3	923 K	12
PbS + 1% Bi_2S_3 + 1% $PbCl_2$	275	-220	13.31	1.1	1.1	923 K	13
PbS + 3% SrS + 2.5% Na	109	290	9.1	0.70	1.2	923 K	14
PbS-Ag 4.4%	**530**	**-177**	**1.66**	**0.82**	**1.7**	**854 K**	**This work**
PbTe-PbS12% + 2% Na	390	252	24	1.1	1.8	800 K	15
$PbTe_{0.7}S_{0.3}$ + 2.5% K	170	294	14.7	0.65	2.0	923 K	16
PbTe + 4% SrTe + 2% Na	285	290	23.9	1.0	2.2	923 K	17
$PbTe_{0.86}PbSe_{0.07}PbS_{0.07}$ + 2% Na	375	270	27.4	1.1	2.0	825 K	18
$(PbSe)_{0.88}(PbS)_{0.12}$ + 0.3% $PbCl_2$	220	260	14	1.1	1.2	900 K	19
$PbTe_{0.1}Se_{0.4}S_{0.5}$-Cl	280	-180	9.1	0.68	0.94	700 K	20

Supplementary Table 4. Electrical conductivity and Seebeck coefficient of PbS-Ag (4.5 mol%) nanocomposite taking into account a weighted sum of properties of the two components.

γ_{PbS}	0.955
γ_{Ag}	0.045
σ_{PbS} (S cm^{-1})	0.1
σ_{Ag} (S cm^{-1})	6.3 x 10^5
S_{PbS} (μV K^{-1})	200
S_{Ag} (μV K^{-1})	6.5
k_{PbS} (W m^{-1} K^{-1})	1
k_{Ag} (W m^{-1} K^{-1})	429
σ_{PbS-Ag} (S cm^{-1})	0.1
S_{PbS-Ag} (μV K^{-1})	200

Supplementary Table 5. Hall charge carrier concentration and mobility measured at ambient temperature.

**Ag content (mol %)**	**Carrier concentration (cm^{-3})**	**Carrier Mobility (cm^2 V^{-1} s^{-1})**
0	p = 1 x 10^{16}	20
1.2	n = 4 x10^{18}	20
4.0	n = 2 x 10^{19}	75
4.4	n = 3 x 10^{19}	90

Supplementary Note 1

Extensive TEM and HRTEM analysis of the nanocomposites was performed. Two main conclusions can be outlined from this analysis:

- Nanocomposites exhibit a highly homogeneous and isotropic microstructure. All regions analyzed showed the same characteristics in all orientations.
- Three distinct crystal phases were observed: 1) relatively large PbS crystal domains; 2) a high density of small nanodomains within the PbS grains and which phase could not be unequivocally identified; 3) elongated nanodomains in between PbS grains identified as *fcc* Ag. The different phases were observed to be homogeneously distributed throughout the nanocomposite.

Supplementary Note 2. Note on figure 2f HRTEM image. When the power spectrum (FFT) for the HRTEM images was obtained, contributions from the Moiré fringes and/or other inclusions that may be present in the sample were included. Therefore the identification of the various phases by inspecting the overall power spectrum was extremely difficult. Since almost all of the observed grains contained many Moiré fringes and some other inclusions, we applied an alternative approach to identify the crystallographic phases. In the image presented in the main text, we filtered the "known" diffraction spots of both *fcc* PbS and *fcc* Ag from the fast Fourier transform (FFT) obtained from the TEM micrograph showing a grain with a ~ 3-4 nm shell-like feature (main text, Fig. 2F). When the "known" diffraction spots of both the *fcc* PbS and *fcc* Ag phases were filtered, it was revealed that the grain consisted of the *fcc* PbS phase covered by a ~3-4 nm Ag shell. These findings are perfectly consistent with the EDX maps obtained from the same sample and the results of the linear combination fits of the XAS data.

Supplementary Discussion

Weighted sum of the properties of two randomly distributed compounds. The electrical conductivity and Seebeck coefficient of a nanocomposite with a random distribution of phases A and B is given by the following equations[6,7]:

$$\text{(Eq. 1)} \qquad \frac{1}{\sigma_{AB}} = \frac{\gamma_A}{\sigma_A} + \frac{\gamma_B}{\sigma_B}$$

$$\text{(Eq. 2)} \qquad S_{AB} = \frac{\frac{S_A\gamma_A}{k_A} + \frac{S_B\gamma_B}{k_B}}{\frac{\gamma_A}{k_A} + \frac{\gamma_B}{k_B}}$$

In eq. 1 and 2, e is the electron charge, γ_A and γ_B are the volume fractions of compounds A and B, respectively.
In the case of PbS-Ag 4.5 mol%, considering a simple weighted sum of the properties, we should obtain nanocomposites with much higher Seebeck coefficients and much lower electrical conductivities as shown in supplementary table 4.

Stability considerations. To ensure thermal stability of the nanocomposites when measured at high temperatures, samples were heated within the LINSEIS system in a He atmosphere up to 850 K at 3 K/min and hold at this temperature for 10 minutes with the boron nitride coating. Such preliminary treatment warranty sample stability for all the cycles tested.
Figure S13 shows the measurements obtained during 3 cycles, including measurements during the stabilization cycle. Notice how measurements obtained during the first temperature ramp are significantly different from the ones obtained in posterior cycles, especially at low temperatures. However, after the first cycle measurements are very repetitive.

To further demonstrate the stability of the material after the first ramp, figure S14 displays the thermoelectric properties of a nanocomposite measured for 5 consecutive up-down cycles between room temperature and up to 850K (after the initial heating up stabilization ramp).

In the low temperature range of the measurement, we observed a slight increase in electrical conductivity with the cycle number and no appreciable variation of the Seebeck coefficient. In the high temperature range of the measurement, a minor decrease of the electrical conductivity with the cycle number (*ca*. -2% after 5 cycles) and an increase of the absolute Seebeck coefficient values (*ca.* +3% after 5 cycles) were obtained. These variations translated in an overall slight increase of the power factor (*ca*. +3% after 5 cycles). Overall we conclude the nanocomposite to have a relatively good stability.

Supplementary methods

Electronic contribution to the thermal conductivity. The electronic contribution to the thermal conductivity (k_{el}) was calculated using the Wiedemann-Franz relation, k_{el}=LTσ, where L is the Lorentz number, T is the temperature, and σ is the electrical conductivity. Usually the Lorentz number is between two limiting values. For metals and highly degenerate electron gases it is $2.45 \cdot 10^{-8}$ Ω W K^{-2}, while the non-degenerate limit is $1.49 \cdot 10^{-8}$ Ω W K^{-2}. A better temperature dependence estimation can be done. The Lorentz number can be estimated based on the Seebeck coefficient measurements and assuming scattering from acoustic phonons, following Fermi-Dirac statistics:

$$S = \pm \frac{k_B}{e}\left[\frac{2F_1(\eta)}{F_0(\eta)} - \eta\right]$$

$$L = \left(\frac{k_B}{e}\right)^2 \left[\frac{3F_0(\eta)F_2(\eta) - 4F_1^2(\eta)}{F_0^2(\eta)}\right]$$

$$F_i(\eta) = \int_0^{\infty} \frac{x^i}{1 + \exp(x - \eta)} dx$$

$$\eta = \frac{E_F}{k_B T}$$

Here, $F_i(\eta)$ are the Fermi-Dirac integrals and k_B is the Boltzmann constant. The values obtained for the Lorentz number are plotted in Supplementary Fig. 8. However, the value used for the calculation of k_{el} and k_{latt} was the non-degenerate limit, due to the special distribution of charge carriers in our nanocomposite.

Porosity correction. In order to study the different contributions to the thermal conductivity, it is necessary to correct thermal and electronic conductivity values for the effects due to porosity of the pellets. An estimation of the electrical and thermal conductivity that would be measured from a 100% dense sample can be obtained using a Maxwell-Eucken expression[1-3]:

$$X_{100} = X_P \frac{1 + \beta P}{1 - P}$$

Here, X_{100} is the electrical or thermal conductivity in the 100% density medium, P is the porosity degree in the range between 0 and 1, and β is an empirical parameter related to the pore geometry, which we fixed to 2[3]. Notice that the thermoelectric figure of merit ZT is not modified by this correction because the porosity effects on the electrical and thermal conductivities compensate for each other.

The Maxwell-Eucken model is one of the most widely used methods to estimate the reduction in conductivity due to porosity. This model is based on randomly distributed spherical pores. The model considers a low concentration of isolated spherical pores. Additionally, the thermal conductivity through the porous medium is considered negligible when compared to that of the solid material. The factor β allows for correction due to pores with a different geometry. If the pores resemble a sphere, the β value is between 1 and 3. Due to the random distribution of pores in our nanomaterials, we estimated a β value of 2[4,5].

Supplementary References

1 Ibáñez, M. *et al.* Core–Shell Nanoparticles As Building Blocks for the Bottom-Up Production of Functional Nanocomposites: PbTe–PbS Thermoelectric Properties. *ACS Nano* **7**, 2573-2586, (2013).

2 Yang, L., Wu, J. S. & Zhang, L. T. Synthesis of Filled Skutterudite Compound $La_{0.75}Fe_3CoSb_{12}$ by Spark Plasma Sintering and Effect of Porosity on Thermoelectric Properties. *J. Alloys Compd.* **364**, 83-88, (2004).

3 Adachi, J., Kurosaki, K., Uno, M. & Yamanaka, S. Effect of Porosity on Thermal and Electrical Properties of Polycrystalline Bulk ZrN Prepared by Spark Plasma Sintering. *J. Alloys Compd.* **432**, 7-10, (2007).

4 Carson, J. K., Lovatt, S. J., Tanner, D. J. & Cleland, A. C. Thermal Conductivity Bounds for Isotropic, Porous Materials. *Int. J. Heat Mass Transfer* **48**, 2150-2158, (2005).

5 Ondracek, G. & Schulz, B. The porosity dependence of the thermal conductivity for nuclear fuels. *J. Nucl. Mater.* **46**, 253-258, (1973).

6 Yu, B. *et al.* Enhancement of Thermoelectric Properties by Modulation-Doping in Silicon Germanium Alloy Nanocomposites. *Nano Lett.* **12**, 2077-2082, (2012).

7 Zebarjadi, M. *et al.* Power Factor Enhancement by Modulation Doping in Bulk Nanocomposites. *Nano Lett.* **11**, 2225-2230, (2011).

8 Razeghi, M. *Fundamentals of Solid State Engineering*. (Springer US, 2009).

9 Knapp, R. A. Photoelectric Properties of Lead Sulfide in the Near and Vacuum Ultraviolet. *Phys. Rev.* **132**, 1891-1897, (1963).

10 Chelvayohan, M. & Mee, C. H. B. Work function measurements on (110), (100) and (111) surfaces of silver. *J. Phys. C: Solid State Phys.* **15**, 2305, (1982).

11 Dweydari, A. W. & Mee, C. H. B. Work function measurements on (100) and (110) surfaces of silver. *Phys. Status Solidi A* **27**, 223-230, (1975).

12 Zhao, L.-D. *et al.* Raising the Thermoelectric Performance of p-Type PbS with Endotaxial Nanostructuring and Valence-Band Offset Engineering Using CdS and ZnS. *J. Am. Chem. Soc.* **134**, 16327-16336, (2012).

13 Zhao, L.-D. *et al.* High Performance Thermoelectrics from Earth-Abundant Materials: Enhanced Figure of Merit in PbS by Second Phase Nanostructures. *J. Am. Chem. Soc.* **133**, 20476-20487, (2011).

14 Zhao, L.-D. *et al.* Thermoelectrics with Earth Abundant Elements: High Performance p-type PbS Nanostructured with SrS and CaS. *J. Am. Chem. Soc.* **134**, 7902-7912, (2012).

15 Girard, S. N. *et al.* High Performance Na-doped PbTe–PbS Thermoelectric Materials: Electronic Density of States Modification and Shape-Controlled Nanostructures. *J. Am. Chem. Soc.* **133**, 16588-16597, (2011).

16 Wu, H. J. *et al.* Broad temperature plateau for thermoelectric figure of merit ZT>2 in phase-separated $PbTe_{0.7}S_{0.3}$. *Nat. Commun.* **5**, 4515, (2014).

17 Biswas, K. *et al.* High-performance bulk thermoelectrics with all-scale hierarchical architectures. *Nature* **489**, 414-418, (2012).

18 Korkosz, R. J. *et al.* High ZT in p-Type $(PbTe)_{1-2x}(PbSe)_x(PbS)_x$ Thermoelectric Materials. *J. Am. Chem. Soc.* **136**, 3225-3237, (2014).

19 Androulakis, J. *et al.* Thermoelectrics from Abundant Chemical Elements: High-Performance Nanostructured PbSe–PbS. *J. Am. Chem. Soc.* **133**, 10920-10927, (2011).

20 Ibáñez, M. *et al.* Electron Doping in Bottom-Up Engineered Thermoelectric Nanomaterials through HCl-Mediated Ligand Displacement. *J. Am. Chem. Soc.* **137**, 4046-4049, (2015).
